# Influence of Corneal Opacity on Intraocular Pressure Assessment in Patients with Lysosomal Storage Diseases

**DOI:** 10.1371/journal.pone.0168698

**Published:** 2017-01-12

**Authors:** Joanna Wasielica-Poslednik, Giuseppe Politino, Irene Schmidtmann, Katrin Lorenz, Katharina Bell, Norbert Pfeiffer, Susanne Pitz

**Affiliations:** 1 Department of Ophthalmology, University Medical Center, Johannes Gutenberg- University Mainz, Germany; 2 Institute for Medical Biostatistics, Epidemiology and Informatics (IMBEI), University Medical Center, Johannes Gutenberg University Mainz, Germany; Xiamen University, CHINA

## Abstract

**Aims:**

To investigate an influence of mucopolysaccharidosis (MPS)- and Morbus Fabry-associated corneal opacities on intraocular pressure (IOP) measurements and to evaluate the concordance of the different tonometry methods.

**Methods:**

25 MPS patients with or without corneal clouding, 25 Fabry patients with cornea verticillata ≥ grade 2 and 25 healthy age matched controls were prospectively included into this study. Outcome measures: Goldmann applanation tonometry (GAT); palpatory assessment of IOP; Goldmann-correlated intraocular pressure (IOPg), corneal-compensated intraocular pressure (IOPcc), corneal resistance factor (CRF) and corneal hysteresis (CH) assessed by Ocular Response Analyzer (ORA); central corneal thickness (CCT) and density assessed with Pentacam. Statistical analysis was performed using linear mixed effect models and Spearman correlation coefficients. The concordance between tonometry methods was assessed using Bland-Altman analysis.

**Results:**

There was no relevant difference between study groups regarding median GAT, IOPg, IOPcc and CCT measurements. The limits of agreement between GAT and IOPcc/IOPg/palpatory IOP in MPS were: [-11.7 to 12.1mmHg], [-8.6 to 15.5 mmHg] and [- 5.4 to 10.1 mmHg] respectively. Limits of agreement were less wide in healthy subjects and Fabry patients. Palpatory IOP was higher in MPS than in healthy controls and Fabry patients. Corneal opacity correlated more strongly with GAT, IOPg, CH, CRF, CCT and corneal density in MPS (r = 0.4, 0.5, 0.5, 0.7, 0.6, 0.6 respectively) than in Fabry patients (r = 0.3, 0.2, -0.03, 0.1, 0.3, -0.2 respectively). In contrast, IOPcc revealed less correlation with corneal opacity than GAT in MPS (r = 0.2 vs. 0.4).

**Conclusions:**

ORA and GAT render less comparable IOP-values in patients suffering from MPS-associated corneal opacity in comparison to Fabry and healthy controls. The IOP seems to be overestimated in opaque MPS-affected corneas. GAT, IOPg and biomechanical parameters of the cornea correlate more strongly with the corneal clouding than IOPcc in MPS patients.

**Trial Registration:**

ClinicalTrials.gov NCT01695161

## Introduction

Mucopolysaccharidoses (MPS) are a group of rare, progressive and chronic lysosomal storage disorders, caused by deficiency of enzymes responsible for degradation of glycosaminoglycans (GAG). The intralysosomal accumulation of interstage GAG-products results in seven MPS types characterized by multimorbidity as well as reduced life expectancy [1]. MPS has a cumulative incidence of 3.5 in 00.000 live births in Germany as demonstrated in a retrospective epidemiological survey analyzing records between 1980 and 1995 [2]. Therefore MPS belong to the orphan diseases. Corneal clouding, glaucoma, ocular hypertension, retinal degeneration and optic nerve swelling or atrophy account for the most frequent ocular complications. Further ocular symptoms such as strabismus, farsightedness, pseudoexophthalmos and hypertelorism have also been reported [3].

The prevalence of glaucoma in MPS patients was assessed at 6.8% [4,5]. In MPS patients glaucoma most possibly is caused by the obstruction of the outflow through the trabecular meshwork [6]. Further mechanisms such as a narrowing of the peripheral chamber angle secondary to a thick iris and peripheral cornea is a matter of discussion [7].

Due to physical as well as mental handicaps in general none of the routinely practiced glaucoma diagnostic methods can be performed properly an reliably in MPS patients as a routine [4,8]. The most common problem is caused by the corneal clouding which impairs the evaluation of the optic nerve head, gonioscopy as well as visual field assessment. In addition slit-lamp examination or applanation tonometry may be impossible due to a short stature or poor cooperation.

The corneal opacity does not only inherit problems concerning the above mentioned examinations, so far also little is known about a possible influence of the corneal opacity on an IOP measurement, which is indispensable for diagnosis and follow-up of glaucoma. In order to determine the effect of corneal opacity on the IOP measurements not only MPS patients will be included in this study but also patients suffering from Morbus Fabry. Fabry disease is a X-linked recessive deficiency or absence of the lysosomal enzyme alpha-galactosidase A. The accumulation of the sphingolipid degradation product globotriaosylceramide (Gb3) within blood vessels, tissue and organs leads to multiply dysfunctions [9]. The Fabry Outcome Survey (FOS) evaluated ocular symptoms in 1203 adult patients suffering from Fabry disease [10]. Cornea verticillata had a similar distribution in women and men and patients with cornea verticillata had a more severe disease versus those without ocular signs. In contrast to MPS, glaucoma is not an overrepresented finding in patients suffering from Morbus Fabry.

Our previous retrospective comparison of rebound tonometry, Perkins applanation tonometry and Ocular Response Analyzer (ORA) in MPS patients showed first hints that corneal opacity could play a role in IOP measurements and demonstrated relevant differences between different tonometry methods. However, corneal-compensated IOP assessed with ORA seemed to be less affected by the MPS-related corneal opacity than applanation tonometry [11]. In this study we aimed to investigate biomechanical properties of the cornea affected from lysosomal storage diseases and their influence on intraocular pressure-measurements in MPS, Fabry patients and healthy controls. Furthermore, we wanted to determine the feasibility of ORA as a non-invasive tonometer in comparison to “gold standard” Goldmann applanation tonometry in MPS patients, and to replicate our previous findings in a prospective study.

## Methods

This observational clinical study was carried out in accordance with the Declaration of Helsinki. Ethics approval was obtained from the Ethics committee of Rhineland-Palatinate, Germany. All MPS and Morbus Fabry patients were transferred from the Department for Lysosomal Storage Disorders of the Children´s Hospital, Mainz University Medical Center. The diagnosis of MPS and Morbus Fabry was confirmed by molecular genetic studies. All patients and volunteers were evaluated between September 2012 and November 2013 at the Department of Ophthalmology University Medical Center of the Johannes Gutenberg University Mainz. Written informed consent was obtained from all participants and/or their parents.

The inclusion criteria for all groups were: ≥ 12 years of age and minimal visual acuity of object fixation. Group 1: MPS type I, II, IV or VI with or without corneal clouding (≥ grade 1). Group 2: Morbus Fabry patients with cornea verticillata ≥ grade 2. Group 3: healthy controls age matched to MPS group.

The exclusion criteria in all groups were: history of refractive surgery, history of corneal transplantation, history of intraocular surgery with exception of uncomplicated cataract surgery longer than 3 months prior to the study visit, inflammation, corneal pathology other than corneal clouding in MPS patients and cornea verticillata in Morbus Fabry.

**Fig 1:**
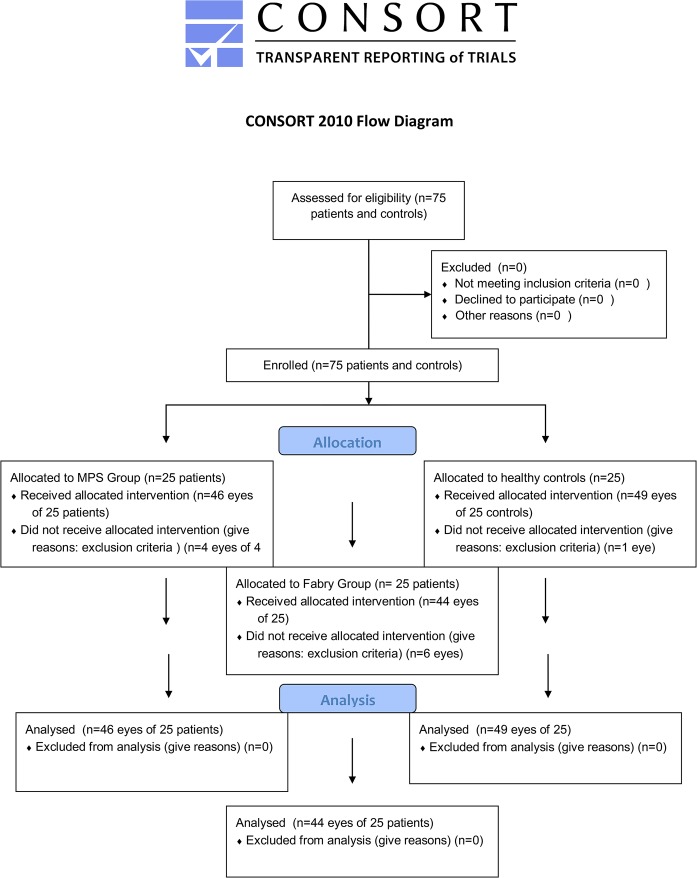
Consort 2010 flow diagram.

The Consort 2010 Flow Diagram is shown in Fig 1.

**Fig 2:**
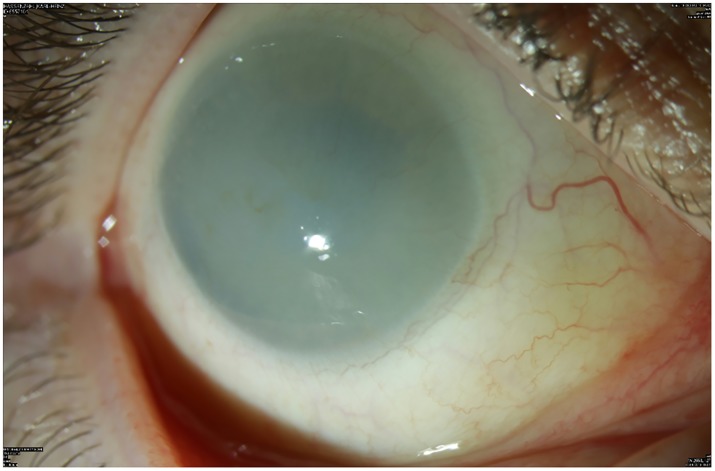
Opaque corneal clouding of grade 4 in patient suffering from MPS I.

All patients underwent an assessment of the best corrected visual acuity (BCVA) with Snellen charts, a slit lamp examination in order to assess a grade of the corneal opacity and central fundus. Grading of corneal clouding in MPS patients was done according to Couprie et al. [12] as follows: grade 1 –no corneal clouding visible; grade 2 –mild corneal clouding, still allowing good visibility of details of the anterior chamber, iris and retina; grade 3 –moderate corneal clouding with partial masking of anterior chamber and iris details as well as reduced fundus view; grade 4 –severe corneal clouding without view on anterior chamber and posterior chamber of the eye (Fig 2).

**Fig 3:**
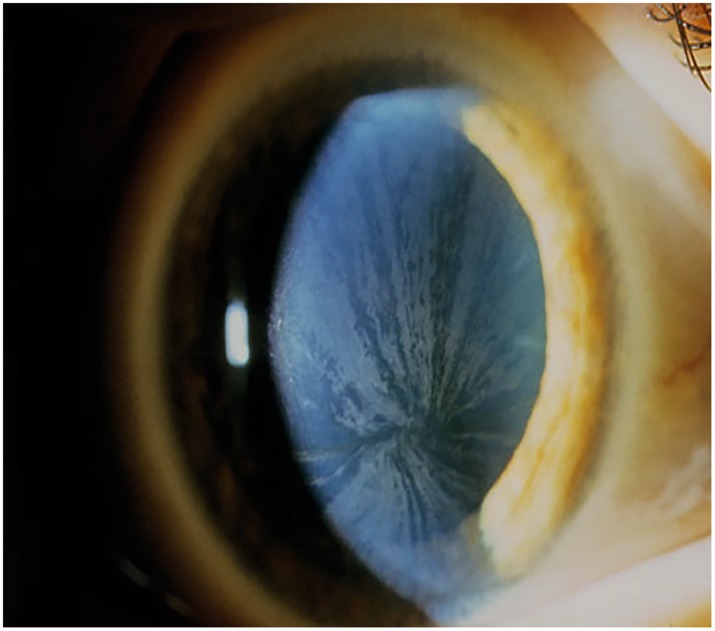
Cornea verticillata grade 3 in patient suffering from Morbus Fabry. A bilateral, whorl-like pattern of powdery, white, yellow or brown corneal deposits.

Grading of cornea verticillata in Morbus Fabry was done according to Orlando scale as follows: grade 1—horizontal line in the inferior third of the cornea; grade 2—arborization (cat’s whiskers); grade 3—a whorl-like pattern; grade 4 –additional clumps of pigment (Fig 3) [13].

All IOP-measurements were assessed in a randomized order. GAT and palpation were performed by two experienced ophthalmologists. ORA- and Pentacam measurements were performed by a medical student, who was experienced and trained in handling of these devices. The GAT-examiners were blinded to the ORA-measurements and to the palpatory IOP-assessment.

The measurement of IOP with GAT [mmHg] requires instilling oxybuprocain-HCl/fluorescein-Na (Thilorbin^®^, OmniVision) eye drops in the lower conjunctival cul-de-sac in both eyes and was performed without pupil dilatation in all cases. The GAT was performed twice. If the difference between measurements was more than 2 mmHg, a third measurement was taken.

The ORA utilizes a visco-elastic structure of the human corneal tissue in a dynamic bi-directional applanation process. The difference in inward and outward pressure values is called corneal hysteresis (CH) [mmHg] and the average of both values provides Goldmann-correlated intraocular pressure (IOPg) [mmHg]. Calculated on the basis of the measured CH, the ORA provides two other parameters: CRF [mmHg] and IOPcc [mmHg], which has been reported to be less affected by corneal properties such as CCT than other tonometry readings [14]. According to our study protocol, three measurements were taken and the value with the best wave-score (ws) was taken for the statistical analysis.

Additionally to the outcome measures planned in the study protocol (supporting file), we assessed the IOP with means of palpation. IOP was assessed by palpation through an ophthalmologist, who had no previous knowledge about the measured GAT- and ORA-IOP. The patients and probands were asked to look down. Redundant skin of the upper eyelid was slightly moved superiorly and ballottement of the globe was performed at the 12 o'clock meridian by applying pressure with each index finger alternately. An assessment of the intraocular pressure was made to the nearest millimeter of mercury.

The CCT [μm] and corneal clouding were assessed with Pentacam^®^. Oculus Pentacam HR Typ 70900 (Fa. Oculus, Weimar, Germany) is a combination of slit illumination and Scheimpflug camera, which rotate together around the eye. Since the cells are not completely transparent, they scatter the light and thus produce a sectional image that is recorded by a side-mounted camera. This camera is oriented in Scheimpflug configuration so that a completely focused image from the anterior corneal surface to the back surface of the lens is created. Corneal density was measured at three points: corneal apex and two peripheral points (3.5 mm to either side of the apex in the 180° axis, at the three and nine o’clock positions). Depending on the degree of light scatter, measurements ranged from 0 to 100, with 0 indicating no light scatter (no corneal haze) and 100 showing a totally opaque cornea. CCT was measured at corneal apex. The average of three measurements was calculated.

## Statistics

The primary objective of this study was to evaluate the concordance of the different tonometry methods. This was done using Bland-Altman analysis. Estimation of sample size was based on the width of the confidence interval for the bias in Bland Altman analysis. With a sample size of 50, a two-sided 95% confidence interval with 95% coverage probability for the bias has an interval that extends no more than 33% of the standard deviation of the bias from the observed bias. For the sample size calculation we assumed that differences between two measurements are independent within patients, therefore 25 patients were recruited. The analysis however, took possible dependencies into account by using linear mixed effect models.

For descriptive analysis means and standard deviations were computed as well as median, 25% and 75% percentiles, minimum and maximum values.

Comparisons between groups of subjects were performed using linear mixed effect models with group as fixed effect and patient / proband as random effect, thereby taking possible dependence between eyes within a subject into account. Influence of corneal clouding was assessed by including corneal clouding as additional fixed effect nested within group. Estimated means and 95% confidence intervals are presented. These analyses are exploratory, therefore p-values are given merely for descriptive purpose and no adjustment for multiple testing was performed.

Spearmann´s correlation coefficient was used to assess correlation between IOP measurements (GAT, IOPcc, IOPg, palpatory assessed IOP) and biomechanical parameters of the cornea (CCT, CH, CRF, density, corneal clouding).

Kruskall-Wallis-test was used to compare non-normally distributed parameters between the three groups. All statistical analyses were performed using SPSS version 22 and SAS version and 9.4.

## Results

### MPS

46 eyes (23 right and 23 left eyes) of 25 MPS patients (12 males, aged mean 29.7, range 14–49 years) were enrolled in this study. Four eyes of 25 MPS patients were not included in the study due to the history of penetrating keratoplasty. This group consisted of 5 (8 eyes) MPS I, 2 (3 eyes) MPS II, 9 (17 eyes) MPS IV, 9 (18 eyes) MPS VI patients. 17 patients (4 MPS I, 2 MPS II, 4 MPS IV and 7 MPS VI) were treated with enzyme replacement therapy (ERT). Most eyes (29) presented the mild corneal clouding of grade 2, followed by 10 patients with the moderate corneal clouding of grade 3. The cornea was clear in 3 eyes (grade 1) and completely opaque in 4 eyes (grade 4).

### Morbus fabry

44 eyes (21 right and 23 left eyes) of 25 Fabry patients (10 males, aged mean 41.6, range 14–73 years) were enrolled in this study. 24 patients were treated with ERT. 24 eyes presented cornea verticillata grade 2 and 20 eyes grade 3.

### Healthy controls

49 eyes (24 right and 25 left eyes) of 25 healthy controls (10 males, aged mean 32.9, range 22–58 years) were enrolled in this study.

The grade of corneal clouding in the right and left eyes in all study groups is presented in Table 1. The disc evaluation was not possible in four eyes of three patients due to MPS-associated corneal opacity grade 4. There was no glaucomatous disc cupping in the fellow—corneal transplanted—eyes of these patients. Three eyes in the MPS group presented swelling of the optic disc. The other patients and probands did not present any glaucomatous disc damage. As glaucoma was not an exclusion criterion in the study, we did not perform further diagnostic like visual field assessment or retinal nerve fiber layer measurement.

**Table 1:** Grade of corneal clouding in the right (OD) and left (OS) eyes in all study groups.

	Grade of the corneal opacity				
	0	1	2	3	4
**Healthy controls OD**	24				
**Healthy controls OS**	25				
**Morbus Fabry OD**			11	10	
**Morbus Fabry OS**			13	10	
**MPS OD**		2	14	5	2
**MPS OS**		1	15	5	2

In Morbus Fabry according to Orlando scale (grade 1—horizontal line in the inferior third of the cornea; grade 2 –arborization, grade 3—a whorl-like pattern, grade 4 –additional clumps of pigment) and in MPS according to Couprie scale (grade 1 –no corneal clouding visible, grade 2 –mild corneal clouding, grade 3 –moderate corneal clouding, grade 4 –severe corneal clouding).

GAT and ORA measurements were performed in all included eyes of the healthy subjects and Fabry patients. In one MPS VI patient GAT could not be performed due to the MPS-related short stature, reduced mobility and poor compliance. The palpatory assessment of IOP was performed in 16 eyes of 8 healthy controls, 21 eyes of 11 Fabry patients and in 33 eyes of 17 MPS patients. Pentacam measurement was performed in all, but one MPS-eye.

Mean values of BCVA (decimal) and spherical equivalent refraction (SEQ) for all study groups are shown in Table 2.

**Table 2:** Mean (estimated from linear mixed effects model) and corresponding 95% confidence interval of BCVA (decimal), spherical equivalent refraction (SEQ) and p-values.

Analysis Variable	Mean Healthy	95% confidence interval, healthy	Mean Fabry	95% confidence interval, Fabry	Mean MPS	95% confidence interval, MPS	p-value
BCVA	1.14	[1.04, 1.24]	1.04	[0.94, 1.14]	0.81	[0.70, 0.91]	< .0001
SEQ	-0.61	[-1.79, 0.57]	-0.67	[-1.98, 0.64]	3.86	[2.64, 5.07]	< .0001

Mean values and 95% confidence intervals of GAT, IOPcc, IOPg, CCT, CH, CRF and corneal density for all study groups are shown in Table 3. Further descriptive statistics by eye is provided as supplementary material (S3 Fig).

**Table 3:** Mean (estimated from linear mixed effects model) and corresponding 95% confidence interval of Goldmann applanation tonometry (GAT), corneal compensated intraocular pressure (IOPcc), Goldmann-correlated intraocular pressure (IOPg), central corneal thickness (CCT), corneal hysteresis (CH), corneal resistance factor (CRF) and corneal density.

Analysis Variable	Mean Healthy	95% confidence interval, healthy	Mean Fabry	95% confidence interval, Fabry	Mean MPS	95% confidence interval, MPS	p-value
IOPcc	14.64	[12.09, 17.20]	14.79	[12.20, 17.37]	16.31	[13.74, 18.88]	0.6005
IOPg	15.25	[12.25, 18.25]	15.75	[12.74, 18.76]	20.07	[17.06, 23.08]	0.0518
GAT	13.75	[12.06, 15.43]	12.99	[11.29, 14.68]	15.78	[14.06, 17.51]	0.0659
CCT	541.00	[516.97, 565.04]	560.49	[536.36, 584.61]	555.14	[530.57, 579.72]	0.5018
CH	11.49	[10.51, 12.46]	11.91	[10.92, 12.90]	13.62	[12.63, 14.60]	0.0076
CRF	11.27	[9.90, 12.65]	12.08	[10.69, 13.46]	14.50	[13.12, 15.87]	0.0043
Dens	24.69	[17.12, 32.25]	31.27	[23.67, 38.88]	57.59	[49.86, 65.33]	< .0001

Without taking corneal clouding into account, GAT, IOPcc and IOPg did not differ between study groups (p>0.05). Palpatory assessed IOP was higher in MPS than in healthy controls and Fabry patients (p<0.05), both in left and right eyes.

CCT did not differ between MPS, Fabry patients and healthy controls (p>0.05). However, CH (p = 0.0076), CRF (p = 0.0043) and density (p < 0.0001) differed between MPS, healthy controls and Fabry.

**Fig 4:**
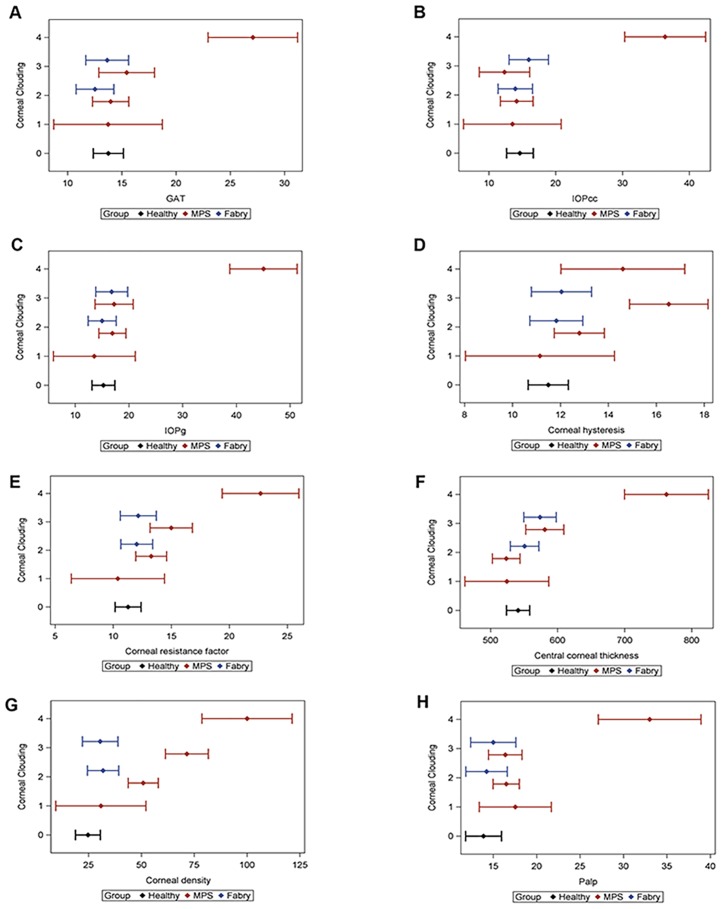
Association of GAT, IOPcc, IOPg, CH, CRF, CCT and density with diagnostic group and the grade of the corneal clouding. Estimated means and 95% confidence intervals are shown.

When taking diagnostic group and corneal opacity into account simultaneously, we found that all GAT, IOPcc, IOPg, CH, CRF, CCT and density all differed and were associated with the grade of the corneal clouding. The results are shown in Fig 4A–4H, the corresponding p-values are displayed in Table 4.

**Table 4:** Effects of diagnostic group and corneal clouding: p-values.

Analysis Variable	Effect	p-value
IOPcc	Group Effect	0.0221
IOPcc	Additional effect of Corneal Clouding	< .0001
IOPg	Group Effect	< .0001
IOPg	Additional effect of Corneal Clouding	< .0001
GAT	Group Effect	0.0008
GAT	Additional effect of Corneal Clouding	< .0001
CCT	Group Effect	0.0016
CCT	Additional effect of Corneal Clouding	< .0001
CH	Group Effect	0.0070
CH	Additional effect of Corneal Clouding	0.0013
CRF	Group Effect	0.0001
CRF	Additional effect of Corneal Clouding	< .0001
Dens	Group Effect	< .0001
Dens	Additional effect of Corneal Clouding	< .0001

**Fig 5:**
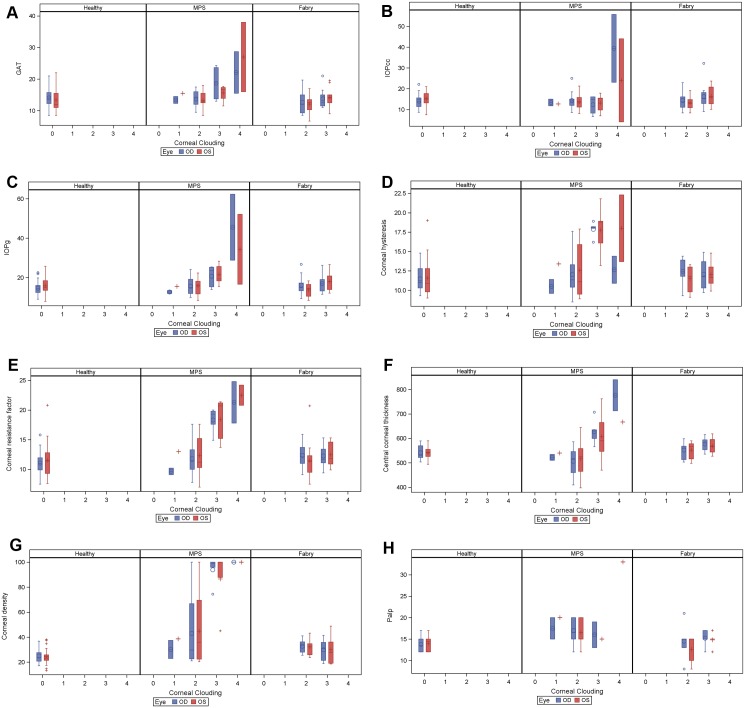
Association between Goldmann applanation tonometry (GAT), corneal compensated IOP (IOPcc), Goldmann correlated IOP (IOPg), corneal hysteresis (CH), corneal resistance factor (CRF) central corneal thickness (CCT), corneal density and the grade of corneal clouding in all experimental groups. Estimated means and 95% confidence intervals are shown.

The Spearman correlation coefficients evaluated between IOP-measurements (GAT, IOPcc, IOPg) and corneal biomechanical parameters (CH, CRF, CCT, density and the grade of corneal opacity) as well as between the grade of corneal opacity and other corneal parameters (CH, CRF, CCT, density) are shown in Table 5 and graphically in Fig 5A–5H. GAT and IOPg correlated more strongly with corneal parameters than IOPcc in the MPS patients. CH, CRF, CCT and density correlated more strongly with the grade of the MPS-associated corneal opacity than with the grade of cornea verticillata.

**Table 5:** The Spearman correlation coefficients evaluated between IOP-measurements: Goldmann applanation tonometry (GAT), corneal compensated intraocular pressure (ccIOP), Goldmann correlated intraocular pressure (IOPg) and corneal biomechanical parameters: central corneal thickness (CCT), corneal hysteresis (CH), corneal resistance factor (CRF), corneal density and the grade of corneal opacity in all study groups.

	Healthy	Fabry	MPS all
**GAT/CH**	0.1	0.1	0.4
**GAT/CRF**	0.3	0.5	0.6
**GAT/CCT**	0.1	0.01	0.4
**GAT/density**	-0.3	0.2	0.5
**GAT/opacity**		0.3	0.4
**IOPcc/CH**	-0.4	-0.4	-0.4
**IOPcc/CRF**	0.1	0.3	0.1
**IOPcc/CCT**	-0.1	-0.2	-0.1
**IOPcc/density**	-0.2	-0.04	0.2
**IOPcc/opacity**		0.3	0.2
**IOPg/CH**	0.2	0.1	0.4
**IOPg/CRF**	0.6	0.7	0.8
**IOPg/CCT**	0.3	0.1	0.5
**IOPg/density**	-0.1	0.1	0.6
**IOPg/opacity**		0.2	0.5
**CH/opacity**		-0,03	0.5
**CRF/opacity**		0.1	0.7
**CCT/opacity**		0.3	0.6
**Density/opacity**		-0,2	0.6

**Fig 6:**
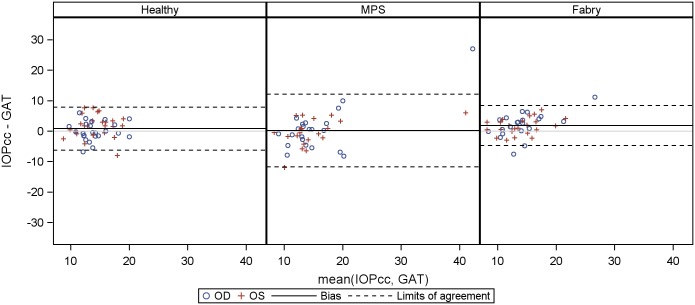
Bland-Altmann Plot for agreement between Goldmann applanation tonometry (GAT) and corneal-compensated IOP (IOPcc).

**Fig 7:**
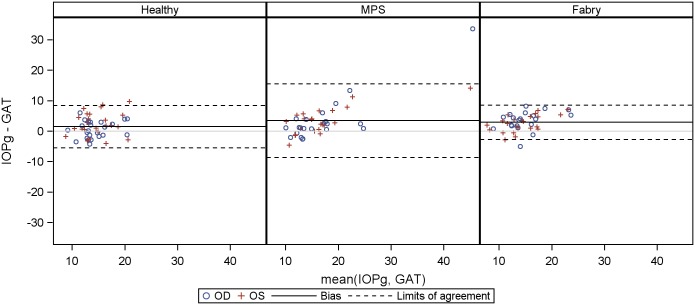
Bland-Altmann Plot for agreement between Goldmann applanation tonometry (GAT) and Goldmann-correlated IOP (IOPg).

**Fig 8:**
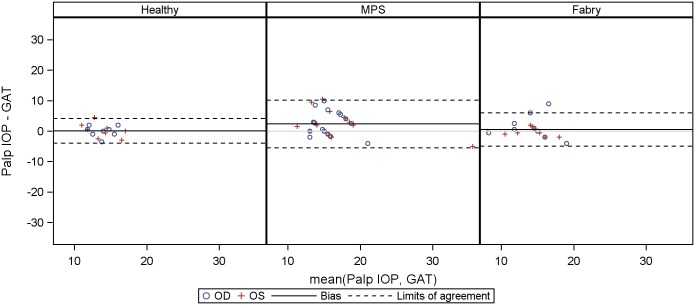
Bland-Altmann Plot for agreement between Goldmann applanation tonometry (GAT) and palpatory assessed IOP (palp IOP).

Agreement between GAT and other tonometry methods (IOPcc, IOPg and palpatory assessed IOP) for healthy controls, Fabry and MPS patients are presented as Bland-Altman plots in Figs 6–8, a table giving a summary can be found in the supplementary material (S3 Fig). The limits of agreement were as wide as [-11.7 mm Hg, 12.1 mmHg] for IOPcc, [-8.6 mmHg, 15.5 mmHg] for IOPg and [- 5.4 mmHg, 10.1 mmHg] for palpatory assessed IOP in MPS patients. Limits of agreement were less wide in healthy subjects and Fabry patients.

### Subgroup analysis of MPS types

Mean values and standard deviations of GAT, IOPcc, IOPg, palpatory assessed IOP, CCT, CH, CRF and corneal density in MPS type I-VI are shown in Table 6.

**Table 6:** Mean values, standard deviations (SD) and p-values of Goldmann applanation tonometry (GAT), corneal compensated intraocular pressure (ccIOP), Goldmann correlated intraocular pressure (IOPg), palpatory assessed IOP (palp. IOP), central corneal thickness (CCT), corneal hysteresis (CH), corneal resistance factor (CRF) and corneal density for the right (OD) and left eyes (OS) in MPS I-IV.

	MPS I	MPS II	MPS IV	MPS VI
**GAT OD**	18,3±7	11,2±1,7	16,6±4,6	13,7±2,6
**GAT OS**	20,9±11,7	9,5	14,3±2,4	13,8±2,8
**IOPcc OD**	23,9±21,8	14,7±0,5	15,5±4,8	13,2±4,1
**IOPcc OS**	21,3±16	14,6	15,5±2,9	10,4±3,3
**IOPg OD**	29±22,3	13,7±0,4	18,4±5,8	16,3±6,5
**IOPg OS**	26,6±17,1	14,8	19,2±5,8	14,7±3
**palp. IOP OD**	20	17,5±3,5	14,8±2,4	17,8±2,3
**palp. IOP OS**	24,3±7,5	20	14±1,3	17,8±2,3
**CCT OD**	627,4±142,1	528,2±25,2	517,9±91,8	567,9±86,4
**CCT OS**	550,3±6,9	541,7	515,2±95,2	572,9±88,4
**CH OD**	13,9±4	10,2±0,9	13,2±3,8	13,6±3,1
**CH OS**	14,3±3,6	11,1	13,8±3,7	15±4,8
**CRF OD**	17,4±5,6	9,7±0,9	13,7±4,4	13,4±4,2
**CRF OS**	17,1±5,4	10,8	14,4±4,7	14,1±4,8
**Density OD**	63,8±39,5	21,9±1,3	76,8±25,3	46,4±33,6
**Density OS**	32,9±10,5	21,1	75,5±23,9	49,3±34

**Fig 9:**
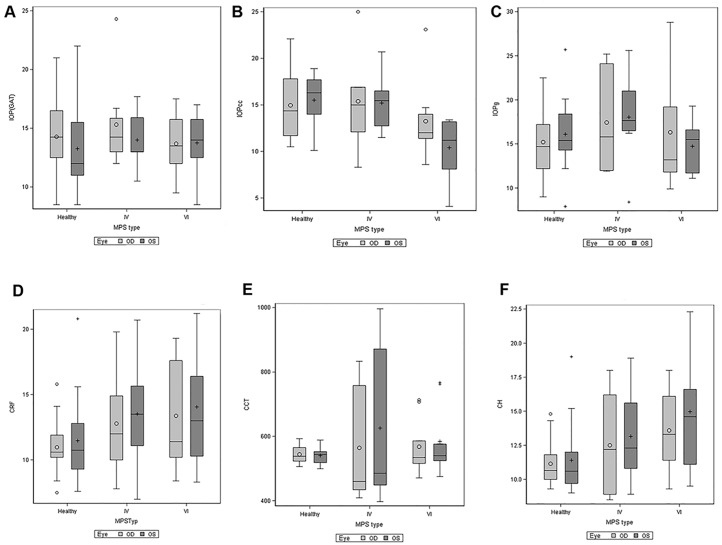
Goldmann applanation tonometry (GAT), corneal compensated IOP (IOPcc), Goldmann-correlated IOP (IOPg), corneal resistance factor (CRF), central corneal thickness (CCT) and corneal hysteresis (CH) in MPS type IV, VI and healthy controls in the right (OD) and the left (OS) eyes.

Due to the higher number of included patients, MPS type IV and VI were included for further statistical evaluation. In the MPS IV group 12 eyes revealed mild and 4 eyes moderate corneal clouding. In the MPS VI group 2 eyes presented clear cornea, 12 eyes mild, 2 eyes moderate and 2 eyes severe corneal clouding. There was no significant difference between study groups regarding GAT-IOP (p = 0.76), IOPg (p = 0.48), CRF (p = 0.14) and CCT (p = 0.5) (Fig 9). CH differed between MPS VI and healthy controls (p = 0.03), but not between MPS IV and other two groups. IOPcc was decreased in MPS VI in comparison to MPS IV (p = 0.02) and healthy controls (p = 0.01).

Corneal clouding in both MPS type IV and VI correlated positively with GAT-IOP, IOPg, CH, CRF, and CCT (r = 0.6, 0.4, 0.7, 0.7, 0.6 in MPS IV and 0.2, 0.6, 0.6, 0.7, 0.7 in MPS VI respectively), but did not correlate with IOPcc (r = -0.1 in MPS IV and r = 0.07 in MPS VI).

## Discussion

The life span and the quality of life of patients with lysosomal storage diseases can be increased due to early diagnosis as well as therapeutic options such as hematopoietic stem cell transplantation or enzyme replacement therapy. These new horizons demand the optimization of diagnostic and therapeutic procedures also in ophthalmology in order to retain a satisfactory visual acuity in these patients for many years. One of the factors leading to reduced visual function in these patients is glaucoma. Therefore we aimed to improve the understanding and management of IOP-measurement in patients suffering from MPS and Morbus Fabry. Our previous retrospective comparison of rebound tonometry, Perkins applanation tonometry and Ocular Response Analyzer (ORA) in MPS patients showed positive correlation between corneal opacity and IOP measurements and demonstrated relevant differences between different tonometry methods [11]. Only ORA-IOPcc seemed to be less affected by the MPS-related corneal clouding. The current study was designed to prospectively evaluate the correlation between IOP measurements and biomechanical properties of the corneas affected by lysosomal storage diseases and to compare feasibility and tolerability of GAT and ORA in MPS patients, Fabry patients and in healthy controls. To the best of our knowledge this is the first prospective study addressing this question.

ORA-measurements were well tolerated by all study patients. In one MPS VI patient GAT could not be performed due to a short stature, difficult positioning at the slit lamp and poor compliance. The proper positioning of some MPS patients was possible only with help of accompanying family members and high motivation of the patients. In the previous study, Perkins applanation tonometry could not be performed in 5 from 17 patients due to reduced compliance (three pediatric patients) and refusal of application of the anesthetic eye drops (two adult patients), whereas ORA measurements were possible in all study patients [11]. In contrast to the current study the previous study performed an analysis of IOP-values collected in the everyday practice and not in the course of a clinical study.

With the mean SEQ of about +3.25 diopters the MPS patients were more hyperopic than Fabry patients and healthy controls. These findings are consistent with those from Ashworth *et al* [5]. Due to the corneal opacity the mean BCVA was as expected the lowest in the MPS group. The Morbus Fabry group was the oldest population of all study groups. The slightly worse BCVA in this cohort is most likely due to some discrete lens opacities and not to the cornea verticillata, which is regarded not to impair the visual acuity [15]. However, worse contrast sensitivity as well as glare problems by normal Snellen visual acuity (black letters on a white background) in Fabry patients was reported in the literature [16].

The global prevalence of glaucoma for population aged 40 to 80 years is 3.54% [17]. In the retrospective case-note review carried out in four tertiary referral centers the prevalence of glaucoma in MPS patients ranged from 2.1% to 12.5% [4]. The limitation of this study was the incomplete diagnostic evaluation of many patients, which reflected the real life problems MPS patients and their ophthalmologists are facing. In our study the disc assessment was possible in 42 eyes of 22 MPS patients. None of them presented glaucomatous disc cupping. As we did not perform further glaucoma diagnostics and could not assess the optic discs in 4 eyes—we cannot specify the prevalence of glaucoma in our MPS group. Neither Fabry patients nor healthy controls presented glaucomatous disc cupping.

The median values of GAT and ORA-IOPs (IOPcc and IOPg) did not differ significantly between MPS, Fabry and healthy controls. However, the limits of agreement between GAT and IOPcc, IOPg and palpatory IOP in MPS were as high as: -11.7 to 12.1mmHg; -8.6 to 15.5 mmHg and—5.4 to 10.1 mmHg respectively. Nevertheless, the maximal values of ORA-IOPs were higher than maximal values of GAT-IOP in Fabry and MPS patients in comparison to controls. This effect was much more pronounced in the MPS group. The extremely high IOP values (GAT of 38 mmHg, IOPcc of 55.7 mmHg and IOPg as high as 62.3 mmHg) were noted in a few MPS patients with a corneal opacity of grade 4. This effect results from changed biomechanical properties of the cloudy corneas and correlates with the grade of the corneal opacity. We speculate that ORA-IOPs, which derive from the viscoelastic corneal property—corneal hysteresis—are more sensitive to strong morphological changes of the cornea than GAT. We conclude that ORA and GAT give more comparable IOP-results in patients suffering from Morbus Fabry than in patients suffering from MPS. The IOP-measurements in strongly affected corneas (grade 4 in MPS) seem to be overestimated, especially with ORA and should be interpreted with caution. On the other hand the palpatory IOP-assessment overestimates the IOP in mild to moderate cases, but is probably more reliable in patients with severe clouding. Palpatory assessed IOP was relevantly higher than other IOP-measurements, but not as high as the maximal values achieved with GAT and ORA in MPS patients with strongly affected corneas. Theoretically, only a direct manometric measurement within the anterior chamber is superior to other tonometry methods and could give an answer to the question, if GAT or ORA represents more real IOP-measurements in the cases of cloudy corneas, but of course it is not practicable in clinical routine [18].

The Spearmann correlation coefficient showed weak correlation between MPS-associated corneal opacity and IOPcc (r = 0.2), but moderate between corneal opacity and GAT or IOPg (r = 0.4 and 0.5 respectively). In our previous study the correlation between IOPcc and the grade of MPS-related corneal opacification was even smaller (r = 0.07) [11]. We explain this discrepancy between our previous and present results with the lower grade of corneal opacity in the population of the previous study (only one of 33 eyes had an opacity grade 4). This lends support to the assumption that the IOPcc is independent of the corneal properties functions in mild to moderate, but not in strongly MPS-affected corneas.

Median CCT as well as median CH and CRF of the right eyes did not differ between study groups. The results are consistent with those from Kottler *et al*. who did not find any significant difference regarding median CCT between MPS II, MPS VI and healthy controls [19]. CH and CRF were relevantly (but *p* close to .05) higher in the left eyes probably due to slightly higher grades of corneal opacity in the left eyes. Corneal density was relevantly higher in MPS and Fabry patients. These results are consistent with those from Elflein *et al*., who also found significantly higher corneal density values in MPS patients in comparison to healthy controls and a strong correlation between the grade of the corneal opacity and the Pentacam parameter [20]. The biomechanical parameter of the cornea: CH, CRF, CCT and corneal density correlated moderately (CH) to strongly (CRF, CCT and density) with the grade of the corneal opacity in the MPS patients and weakly in the Fabry group. In the pediatric study Connel *et al*. showed a similar correlation between CCT and MPS I-related corneal opacity (r = 0.57) [21]. Our data show a trend, and supports the assumption that accumulation of GAG in the ocular tissue changes its biomechanical features and hence leads to increased CH and CRF values. Our results are consistent with a study of Fahnehjelm *et al*., which reported increased CH and CRF in MPS [22].

In summary, ORA and GAT seem to render less comparable IOP-results in patients suffering from MPS in comparison to Morbus Fabry and healthy controls. IOP-measurements in strongly affected corneas seem to be overestimated and should be interpreted with caution. In these cases palpatory IOP-assessment could be helpful. Due to relatively high limits of agreement between different tonometry methods, it seems to be important to use the same, individually adjusted and well-tolerated device for the follow-up of individual patients. On the other hand a constant worsening of the MPS-related corneal opacity may demand a change of the tonometry method.

## Supporting Information

S1 Fig Study protocol. (DOC)

S2 Fig Trendstatement_trend_checklist. (DOCX)

S3 Fig Descriptive statistics for BCVA, SEQ, GAT, IOPcc, IOPg, IOPpalp, CCT, CH, CRF and corneal density for all study groups(Table S1 and S2); summary of Bland Altman analyses (Table S3). (DOCX)

S4 Fig Raw data and statistical analysis software (SAS). (ZIP)
