# Supplementary material for: Influence of Corneal Opacity on Intraocular Pressure Assessment in Patients with Lysosomal Storage Diseases
Source: PLoS One. 2017 Jan 12;12(1):e0168698. doi: 10.1371/journal.pone.0168698 (PMC5230782; doi:10.1371/journal.pone.0168698)
Supplement: S1 Fig — (DOC) [file pone.0168698.s001.doc]

**ORA in MPS**

**Non-invasive Assessment of Intraocular Pressure in MPS by Use of the Ocular**

**Response Analyzer – Optimization of Routine Ophthalmological MPS Patient**

**Care**

**Corneal-Compensated Intraocular Pressure, Corneal Hysteresis and Corneal Resistance Factor Measurements Performed with the Ocular Response Analyzer and their correlation to conventional Goldmann Applanation Tonometry – new Horizons in non-invasive Glaucoma Diagnostic in Mucopolysaccharidosis?**

**Sponsor:**

Dep. of Ophthalmology, University Medical Center, Johannes Gutenberg-University Mainz, Germany

**Principal Investigator**:

Name: Susanne Pitz, MD PhD

Address: Department of Ophthalmology, University Medical Center, Johannes Gutenberg-

University Mainz, Langenbeckstr. 1, 55131 Mainz, Germany

Phone +49 6131 176762

Fax +49 6131 173455

e-Mail: Susanne.Pitz@unimedizin-mainz.de

**Co-Investigators**: Joanna Wasielica-Poslednik, MD, Giuseppe Politino, Katharina Bell, MD, Katrin Lorenz, MD

**Study coordinator**: Joanna Wasielica-Poslednik, MD

**Statistician:** Irene Schmidtmann

**Synopsis**

| **Title** | Non-invasive Assessment of Intraocular Pressure in  Mucopolysaccharidosis (MPS) by Use of the Ocular Response Analyzer –Optimization of Routine Ophthalmological MPS Patient Care |
| --- | --- |
| **Short Title** | ORA in MPS |
| **Protocol No.** | MZ-MPS-2012-03 |
| **Sponsor** | Dep. of Ophthalmology, University Medical Center, Johannes Gutenberg-University Mainz, Germany |
| **Principal Investigator** | Susanne Pitz MD, PhD |
| **Study Design** | Comparative case-control study |
| **Patient population** | MPS I, II, IV and VI |
| **Sample size** | 25 MPS patients  25 Fabry patients  25 healthy age matched controls (a difference of ± 5 years in the mean between both groups is accepted) |
| **Inclusion Criteria** | MPS I, II, IV, VI patients with grade 1-4 of corneal clouding (Couprie et al.)  Fabry patients with cornea verticillata > grade 1  Age ≥ 12 years  Patient is able to comply with the study procedure  Patient has consented to be in the trial  Ability to fixate a target |
| **Exclusion Criteria** | History of corneal transplantation or refractive surgery  Corneal pathologies other than MPS-associated corneal opacity  Corneal, conjunctival or intraocular inflammation |
| **Objectives** | *Primary objective*   - To determine feasibility of ocular response analyzer as non-invasive intraocular pressure-measurement-device in comparison to “gold standard” Goldmann applanation tonometry in MPS patients   *Secondary objective*   - To investigate biomechanical properties of the cornea and their influence on intraocular pressure-measurements in MPS, Fabry and healthy controls   *Tertiary objective*   - To investigate differences between MPS-subtypes regarding corneal properties and their influence on tonometry readings |
| **Investigational Medicinal Product** | - Ocular Response Analyzer (ORA) - Goldmann Applanation Tonometry (GAT) - Pentacam |
| **Methods/ Study procedures** | - Best corrected visual acuity (BCVA): obtained by Snellen charts - Slit lamp microscopy of anterior segment: grading of corneal clouding, exclusion of other corneal pathologies - Indirect ophthalmoscopy with undilated pupil: optic nerve assessment - ORA measurements: corneal compensated intraocular pressure (ccIOP), corneal hysteresis (CH) and corneal resistence factor (CRF) - GAT - Pentacam: assessement of the corneal clouding and corneal thickness |
| **Endpoints** | *Primary Endpoint:*   - Evaluation of agreement between ccIOP, IOPg and GAT in MPS, Fabry and healthy controls   *Secondary Endpoint*   - Evaluation of biomechanical properties of the cornea (CH and CRF measured by ORA) and their correlation with intraocular pressure measurements (ccIOP and GAT) in MPS, Fabry and healthy controls - Evaluation of corneal clouding and corneal thickness (CCT) by use of Pentacam in MPS, Fabry and healthy controls - Correlation between corneal clouding, CCT and ccIOP/GAT   *Tertiary endpoint*   - Comparison between MPS-subtypes regarding ccIOP, CH, CRF, GAT, corneal density and CCT |
| **Trial Duration and Dates** | Enrolment and evaluation period of about 1 year |

**Prof. Susanne Pitz**

**Observational Research Project: Non-invasive assessment of intraocular pressure in MPS by use of the ocular response analyzer – optimization of routine ophthalmological MPS patient care**

**Background**

Glaucoma is a condition leading to progressive optic nerve damage and corresponding defects in the affected person’s visual field. The main causative factor so far identified is elevated intraocular pressure (IOP). Though other factors (blood flow, family history, myopia, race) also may play a role, IOP is the best characterized one. It is widely accepted that an IOP of 21 mm Hg or below is “normal”, while values above are related to a considerable risk to develop glaucomatous optic nerve damage. Thus, measurement of IOP is one of the most important diagnostic measures in glaucoma, and lowering of IOP represents the major therapeutic approach.

Glaucoma is one of the known ocular complications of the mucopolysaccharidoses (MPS). The main underlying pathomechanism is thought to be the accumulation of glycosaminoglycans (GAGs) in the anterior segment of the eye (McDonnell et al, 1985; Stürmer, 1989; Iwamoto et al, 1990). This results in a situation quite similar to what is termed “open angle glaucoma”: It is characterised by a normal production of aqueous humour, but impaired outflow via the trabecular meshwork. In MPS, the progressive deposition of GAGs within this structure leads to an increased outflow resistance. It may occur in any MPS type, but seems to be more frequent in MPS I, IV and VI, and has been reported to range as high as 40% (Ashworth, 2006 a, b).

Due to the specific problems of MPS patients, the accurate diagnosis of glaucoma is hampered by a number of factors, one of them being the corneal clouding. This impairs the visualization of the optic nerve (which is the site of potential damage of the elevated pressure) and the anterior chamber angle (the outflow site of aqueous humour). As corneal clouding reduces visual acuity, it also impairs the patient’s ability to cooperate in visual field testing, which is the most important diagnostic tool to assess optic nerve function. Moreover, the patient’s ability to cooperate in this test may be limited due to young age, mental status and short stature, the latter in many cases not allowing a comfortable positioning during examination. Cooperation also is critical for virtually all other examinations: slit lamp investigation and applanation tonometry all require stable positioning and acceptance of bright light and – for tonometry – application of anaesthetic eye drops. Thus, in clinical routine, the spectrum of examinations performed to rule out glaucoma in an MPS patient is often far from what is routinely performed in standard patient care.

In addition to all these obstacles, MPS corneas are not “normal”. Like other ocular tissues the cornea has been shown on the histological level to be affected by the storage process (McDonnell et al 1985). It is well established that the posterior sclera is significantly thickened in MPS (Beck and Cole 1984, Schumacher et al 2008), however there is a long-standing controversy regarding corneal thickness (Stürmer 1989; Iwamoto et al 1990; Ashworth et al 2006 b), and it seems reasonable to assume that the biomechanical properties differ form normal. Deviation from “normal” corneal thickness is regarded as one of the most important sources of measurement errors in Goldmann applanation tonometry (Whitacre and Stein, 1993).

Corneal thickness in MPS and its correlation to corneal clouding and IOP has been investigated in two recent studies. Connell and co-workers found a correlation between corneal clouding, corneal thickness and IOP, suggesting that the cloudier an MPS-cornea, the more likely to be thicker and to cause overestimation of IOP as measured by tonometry. However, this study was hampered by the use of diverse IOP measurement techniques and different examination conditions as some values were obtained in awake patients, others under general anaesthesia (which is known to lower the IOP, depending on duration and depth of anaesthesia). To the opposite, Kottler et al (2010) could not confirm a correlation of IOP and corneal thickness or an overall increased corneal thickness in their cohort of 28 MPS II and VI patients, suggesting that applanation tonometry seems a reliable method in MPS patients. In summary, there is considerable debate on the accuracy of applanation tonometry in this setting.

Up to now, almost nothing is known about the biomechanical properties of the cornea in MPS. As the storage process is known to significantly involve sclera and cornea, it seems likely that these properties differ from normal in the MPS eye. This is of great relevance, as applanation tonometry implies “normal” tissue properties (Whitacre and Stein, 1993). A novel technical development addressing this problem is to measure the IOP corrected for corneal biomechanical properties. This recently became possible by the Ocular response analyzer (ORA, Reichert Inc., Buffalo, NY). This device simultaneously measures the intraocular pressure as well as a number of corneal tissue features, such as corneal hysteresis, which is a measure of the viscoelasticity (most likely not only of the cornea but also of the sclera, as both form an anatomic unit) and corneal resistance. The ORA monitors the corneal response to a 20 ms air puff by an electro-optical collimation detector system. This response consists in an initial inward, and then outward movement, during which two applanation events are measured. The difference between “inward” and “outward” applanation depends on the viscoelasticity and is expressed as corneal hysteresis. This novel technology is the only one enabling the investigation corneal tissue features and currently is applied to characterize different corneal disorders such as corneal dystrophies or corneas after refractive surgery (Chihara, 2008). It has been reported to have a good reproducibility (Wasielica-Poslednik, 2010). In addition to hysteresis, IOP, a “corneal compensated IOP” (IOPcc; postulated to be independent from corneal thickness) and the corneal resistance factor CRF are measured.

In a recent study, Fahnehjelm et al found an increased corneal hysteresis in 12 eyes of 7 MPS I and VI patients (Fahnehjelm et al, 2011). Their results lend support to the assumption that this leads to a false too high IOP readings when measure by conventional applanation tonometry. However, their study may be criticised for a number of shortcomings: small sample size, unspecified method of intraocular pressure measurement and lacking data of corneal thickness (Gatzioufas et al, 2011).

In an own pilot study, we had the opportunity to gather some experience with the ORA in our out patient clinic for MPS patients (the ORA was kindly put at our disposal by Eyetec GmbH, Lübeck, Germany). Within a 2-week period, we were able to measure 27 eyes of 14 MPS patients. While applanation tonometry was not possible in 5 patients due to their refusal of the local anaesthetic, all but one patient underwent the ORA measurement without problems and reported it to be easily tolerable. Two factors seemed important for the good patient acceptance: the measurement is a non-contact procedure without need of administration of eye drops, and it is very quick, requiring only a few seconds of head positioning at the chin rest of the device. Additionally, despite short stature, our MPS patients had less problems to sit (or stand) properly at the ORA in comparison to the slit lamp.

In view of these preliminary experiences, we are confident that the proposed project will be feasible as well in terms of patient cooperation, as in terms of sample size.

**Aim of the study**

The aim of the present study is to investigate ccIOP and corneal hysteresis using the ORA in a group of MPS patients, and to compare it to the classical gold standard of IOP measurement, to Goldmann applanation tonometry. Regression-based Bland and Altman analysis will be used to evaluate agreement between the instruments.

Additionally a possible correlation to corneal thickness and to an automated quantification of corneal clouding by the Scheimpflug principle will be investigated. The Pentacam is a device allowing a number of diagnostic investigations (topography, measurement of anterior and posterior corneal surface, corneal reflectivity, thickness) related to the corneal anatomy and pathology by means of a rotating Scheimpflug imaging system. During a non-contact procedure, 50 images are taken within 2 seconds of measurement duration. We were able to confirm the feasibility of Pentacam measurements to judge corneal clouding (personal oberservation, unpublished data).

We hypothesize that this spectrum of methods enables a more reliable judgement of IOP an MPS patients, as all these parameters are known or speculated to influence IOP readings. Ideally, the results will allow a more precise assessment of the prevalence of glaucoma in the diverse MPS types.

A weakness of this research proposal is one that is one typically inherent to studies investigating the IOP: Goldmann applanation tonometry is regarded to be the current gold standard, despite a huge number of possible measurement errors (Whitacre and Stein, 1993). As the cornea (and sclera) of MPS patients are very likely to differ from normal, the measurement errors can be expected to be even greater. Nevertheless, there is no other “gold standard” available. Theoretically, a direct manometric measurement within the anterior chamber is superior to applanation tonometry, however is not feasible due to its invasive nature. This in consequence means that the ORA measurements are likely to be compared to a “weak” gold standard, as applanation tonometry may be less reliable in MPS patients than in healthy subjects. It may – at least with non-invasive techniques - be virtually impossible to prove, what the true IOP in an MPS patient is. Despite all these limitations, the investigation of diseased corneas by means of the ORA seems the only currently available technique, which provides information on corneal tissue properties.

This weakness may at least partially be compensated by a specific study design, including two control groups: As control group, age and gender matched normal individuals will undergo ORA measurements.

Additionally, another group of patients regularly seen out our out patient clinic for patients suffering from lysosomal storage disorders, Fabry patients, will be included as a second control group. Fabry disease is well known to show a very distinctive corneal opacity, cornea verticillata (Sher et al, 1979; Sodi et al 2007). It seems of interest to investigate corneal hysteresis in this disorder, which is regarded to have normal biomechanical characteristics, and no increased risk for glaucoma. On the other hand, to the best of our knowledge cornea verticillata so far has not been studied in respect of corneal hysteresis. As the majority of corneal dystrophies up to now have been found to exhibit a decreased corneal hysteresis (Kotecha, 2007), the difference in corneal hysteresis to be detected between MPS and Fabry corneas should be even greater and would therefore underline the significance of the results.

To date, up to 40% of MPS patients are regarded to suffer from glaucoma (Ashworth 2006 a, b). As the investigations needed to establish this diagnosis are substantially hampered by the typical ophthalmological complications of the MPS disorders, diagnosis – and consequently treatment – is often based on IOP readings only. Depending on the findings of the proposed research project, it seems reasonable to assume that ORA measurements may improve standard ophthalmological care for MPS patients, as the method has the potential to give additional information enabling a more precise assessment of the IOP. Thus, unnecessary diagnostic procedures and overtreatment might be avoided in future, contributing to an increased quality of life of the affected patients.

**Study design**

Comparative case-control study.

**Methods**

Visual acuity: will be obtained by the Snellen charts with best correction /pinhole.

ORA measurement: will be performed by a different observer than applanation tonometry.

Anterior segment investigation: done by slit lamp microscopy. Grading of corneal clouding will be done according to Couprie et al, 2010:

1: no corneal clouding visible

2: subtle corneal clouding, still allowing good visibility of details of the anterior chamber/iris structure and retina

3: corneal clouding with partial masking of anterior chamber/iris details and reduced fundus view

4: severe corneal clouding without view on anterior/posterior chamber of the eye.

Assessment of anterior chamber depth will be done according to van Herick (van Herick et al, 1969).

Funduscopy/optic nerve assessment: done with undilated pupil as indirect ophthalmoscopy, preferably by means of a +78/+90 dpt lens at the slit lamp, or using a + 20 dpt lens in cases of poor cooperation. Optic nerve assessment will comprise estimation of the cup/disc ratio, assessment of the distribution of the nerve fibres at the disc margin (ISNT rule), eventual non glaucoma-related optic disc pallor or swelling or any other optic disc pathology.

Applanation tonometry: Applanation tonometry will be carried out by an observer unaware of the ORA readings.

The tonometer pressure will be set at 10 mm Hg prior to measurement for each patient. This measure should avoid an overestimation of IOP which is known to result from a too low setting of IOP at the tonometer, allowing an increased indentation of the globe.

After instillation of a fixed oxybuprocain 0.4%/fluorecscein 0.08% combination, the IOP will be measured three consecutive times within a few seconds in the right eye, then three consecutive times in the left eye. After comfortable positioning at the slit lamp, the patients are asked to fixate a distant target (5m) in order to rule any affect of accommodation on IOP. The first measurement of the right eye will be discarded, as it usually ranges significantly higher than the following readings. Though this sequential measurement is known to result in lowering the IOP due to the tonographic effect, it is recommended for specific settings: it gives the least range of IOP values per eye (Whitacre and Stein, 1993). This seems desirable in the context of this investigation, as the applanation values are to be compared to the ORA measurements.

Scheimpflug investigation of the cornea and measurement of corneal thickness (Pentacam):

Simultaneously, corneal density, a measure for opacification ranging from 0 and 100 (0 = clear cornea; 100 = tissue completely opaque) and corneal thickness (measurement of the anterior and posterior corneal surface) will be measured in a non contact procedure.

The investigations will be carried out by a team of ophthalmologists, orthoptists and technicians highly experienced in the care of MPS patients, familiar with the specific problems of ophthalmological examinations in this group of patients with often reduced cooperation. Besides the long-standing expertise regarding the ophthalmological changes in lysosomal storage disorders (Pitz et al, 2007, 2009; Schumacher et al, 2008; Kottler et al, 2010; Wasielica-Poslednik 2011) a main scientific focus of our department is glaucoma research (Barleon et al, 2006; Bourne et al, 2007; Hoffmann et al, 2007a, b; Schiefer, 2009; Wasielica-Poslednik et al 2010; Schulze et al, 2011). Thus, we are confident not only to provide adequate standard in the investigations, but also sufficient scientific background to deliver appropriate interpretation of the data obtained.

**Sample size considerations**

In the pilot study Fahnehjelm et al (Fahnehjelm et al, 2011) reported a corneal hysteresis of mean 16.6 in 12 eyes of 7 MPS patients in comparison to 11.7 (standard deviation: 1.1) as reported for this age group (Ortiz et al, 2007). These data allow the following sample size calculation, which is based on slightly more “pessimistic” figures than the data from literature.

Estimation of sample size was based on two group Satterthwaite t-test of equal means (unequal variances) for the primary efficacy criterion, i.e. the difference in corneal hysteresis between the study groups. A sample size of 25 in each group will have 90% power to detect a difference in means of -3.0 (the difference between a group 1 mean of 11.5 and a group 2 mean of 14.5) assuming that group 1 standard deviation is 2.0 and the group 2 standard deviation is 4.0 (ratio of group 2 to group 1 standard deviation is 2.0) using a two group Satterthwaite t-test with a 0.05 two-sided significance level (Moser et al. 1989).

To assess an agreement between Goldmann applanation tonometry and ORA, regression-based Bland and Altmann analysis will be used. Due to the fact that differences between IOPcc and IOPg reported in the study of Fahnehjelm et al (2011) varied strongly than SD for corneal hysteresis in our sample size calculation, we assume that our estimated sample size will be adequate for IOP measurements either.

This number of patients seems realistically to include within a reasonable time frame, when considering the number of patients seen at our outpatient clinic of MPS patients (MPS types I, II, IV and VI), where we approximately see 2 MPS patients per week. Assuming that 25% of these are willing and able to participate, this sample size should be reached within one year. This hold even more true in view of the fact that during our own pilot project we were able to measure 14 MPS patients with the ORA within two weeks.

As control group, age and gender matched normal individuals will undergo ORA measurements. Additionally, another group of patients regularly seen out our out patient clinic for patients suffering from lysosomal storage disorders, Fabry patients, will be included as a second control group. Fabry disease is well known to show a very distinctive corneal opacity, cornea verticillata (Sher et al, 1979; Sodi et al 2007). As corneal dystrophies so far have been mainly reported to have a reduced corneal hysteresis, the difference of hysteresis values between patients suffering from these two lysosomal storage disorders should be greater than between MPS patients and normal individuals.

**Literature:**

Ashworth JL, Biswas S, Wraith E, Lloyd IC. The ocular features of the mucopolysaccharidoses. Eye 2006 a;20:553-563.

Ashworth JL, Biswas S, Wraith E, Lloyd IC. Mucopolysaccharidoses and the eye. Surv Ophthalmol 2006 b;51:1-17.

Barleon L, Hoffmann EM, Berres M, Pfeiffer N, Grus FH. [Comparison of dynamic contour tonometry and goldmann applanation tonometry in glaucoma patients and healthy subjects.](http://www.ncbi.nlm.nih.gov/pubmed/17011849) Am J Ophthalmol. 2006;142:583-90.

Beck M, Cole G. Disc oedema in association with Hunter’s syndrome: ocular histopathological findings. Br J Ophthalmol 1984;68:590-594.

Bourne RR, Jahanbakhsh K, Boden C, Zangwill LM, Hoffmann EM, Medeiros FA, Weinreb RN, Sample PA. [Reproducibility of visual field end point criteria for standard automated perimetry, full-threshold, and Swedish interactive thresholding algorithm strategies: diagnostic innovations in glaucoma study.](http://www.ncbi.nlm.nih.gov/pubmed/17919445) Am J Ophthalmol. 2007;144:908-913.

Chihara E. Assessment of true intraocular pressure: the gap between theory and practical data. Surv Ophthalmol 2008;l53:203-218.

Connell P, McCreery K, Doyle A, Darcy F, O’Meara A, Brosnahan D. Central corneal thickness and its relationship to intraocular pressure in mucoplosaccharidoses-1 following bone marrow transplantation. J AAPOS 2008;12:7-10.

Couprie J, Denis P, Guffon N, Reynes N, Masset H, Beby F. Manifestations ophthalmologiques de la maladie de Morquio. J Fr Ophthalmol 2010;33:617-622.

Fahnehjelm KT, Chen E, Winiarski J. Corneal hysteresis in mucopolysaccharidosis I and VI. Acta Ophthalmologica 2011; doi10.1111/j.1755-3768.2010.02085.x

Gatzioufas Z, Schirra F, Seitz B. Corneal hysteresis in mucopolysaccharidosis I and VI. Acta Ophthalmol. 2011 Sep 28. doi: 10.1111/j.1755-3768.2011.02245.x.

Hoffmann EM, Boden C, Zangwill LM, Bowd C, Medeiros FA, Crowston JG, Sample PA, Weinreb RN. [Intereye spatial relationship of abnormal neuroretinal rim locations in glaucoma patients from the diagnostic innovations in glaucoma study.](http://www.ncbi.nlm.nih.gov/pubmed/17379176) Am J Ophthalmol 2007;143:781-7.

Hoffmann EM, Zangwill LM, Crowston JG, Weinreb RN. [Optic disk size and glaucoma.](http://www.ncbi.nlm.nih.gov/pubmed/17212989) Surv Ophthalmol. 2007 Jan-Feb;52(1):32-49.

Iwamoto M, Nawa Y, Maumenee IH, Young-Ramsaran J, Matalon, R, Green WR. Ocular histopathology and ultrastructure of Morquio syndrome (systemic mucopolysaccaridosis IV A). Graefes Arch Clin Exp Ophthalmol 1990;228:342-349.

Kotecha A. What biomechanical properties of the cornea are relevant for the clinician? Surv Ophthalmol 2007;52:109-114.

Kottler U, Demir D, Schmidtmann I, Beck M, Pitz S. Central corneal thickness in mucopolysaccharidoses II and VI. Cornea 2010;29:260-262.

McDonnell J, Green WR, Maumenee IH. Ocular histopathology of systemic mucopolysaccharidosis, type II-A (Hunter syndrome, severe). Ophthalmology 1985;92:1772-1779.

Moser BK, Stevens GR, Watts CL. The two-sample t test versus Satterthwaite´s approximate F test. Commun Statist-Theory Meth 1989;18: 3963-3975.

Pitz S, Ogun O, Arash L, Miebach E, Beck M. [Does enzyme replacement therapy influence the ocular changes in type VI mucopolysaccharidosis?](http://www.ncbi.nlm.nih.gov/pubmed/19159944) Graefes Arch Clin Exp Ophthalmol. 2009;247:975-980.

Pitz S, Ogun O, Bajbouj M, Arash L, Schulze-Frenking G, Beck M. [Ocular changes in patients with mucopolysaccharidosis I receiving enzyme replacement therapy: a 4-year experience.](http://www.ncbi.nlm.nih.gov/pubmed/17923542) Arch Ophthalmol 2007;125:1353-1356.

Schiefer U, Pascual JP, Edmunds B, Feudner E, Hoffmann EM, Johnson CA, Lagrèze WA, Pfeiffer N, Sample PA, Staubach F, Weleber RG, Vonthein R, Krapp E, Paetzold J. [Comparison of the new perimetric GATE strategy with conventional full-threshold and SITA standard strategies.](http://www.ncbi.nlm.nih.gov/pubmed/19060285)Invest Ophthalmol Vis Sci. 2009;50:488-494.

Schulze A, Lamparter J, Pfeiffer N, Berisha F, Schmidtmann I, Hoffmann EM. [Diagnostic ability of retinal ganglion cell complex, retinal nerve fiber layer, and optic nerve head measurements by Fourier-domain optical coherence tomography.](http://www.ncbi.nlm.nih.gov/pubmed/21240522) Graefes Arch Clin Exp Ophthalmol. 2011 Jan 15.

Schumacher RG, Brzezinska R, Schulze-Frenking G, Pitz S. [Sonographic ocular findings in patients with mucopolysaccharidoses I, II and VI.](http://www.ncbi.nlm.nih.gov/pubmed/18299822) Pediatr Radiol 2008;38:543-550.

Stürmer J. Mukopolysaccharidose Typ VI-A (Morbus Maroteaux-Lamy). Klinisch-pathologischer fallbericht. Klin Mbl Augenheilk 1989;194:273-281.

Wasielica-Poslednik J, Berisha F, Aliyeva S, Pfeiffer N, Hoffmann EM. Reproducibility of ocular response analyzer measurements and their correlation with central corneal thickness. Graefes Arch Clin Exp Ophthalmol 2010;DOI 10.1007/s00417-010-1471-1

Wasielica-Poslednik J, Pfeiffer N, Reinke J, Pitz S. Confocal laser-scanning microscopy allows differentiation between Fabry disease and amiodarone-induced keratopathy. Graefes Arch Clin Exp Ophthalmol 2011;249:1689-1696.

Whitacre MW, Stein R. Sources of error with use of Goldmann-type tonometers. Surv Ophthalmol 1993;38:1-30.

van Herick W, Shaffer RN, Schwartz A. Estimation of width of angle of anterior chamber. Incidence and significance of the narrow angle. Am J Ophthalmol 1969;68:626 –629.

**Data handling and record keeping/ CRF**

Paper case report forms and according work sheets will be developed by the study coordinator before the study starts

Informed consent

**Ethical approval**

**Informed consent**

Written informed consent will be obtained from each patient before any study-specific procedures will be performed

**Clinicaltrials.gov**

The study will be registered at clinicaltrials.gov before the first patient will be included in the trial.
