# Supplementary material for: Influence of Corneal Opacity on Intraocular Pressure Assessment in Patients with Lysosomal Storage Diseases
Source: PLoS One. 2017 Jan 12;12(1):e0168698. doi: 10.1371/journal.pone.0168698 (PMC5230782; doi:10.1371/journal.pone.0168698)
Supplement: S3 Fig — (DOCX) [file pone.0168698.s003.docx]

# Supplementary Material

|  | | | **Mean** | **Std** | **95% CI, lower limit** | **95% CI, upper limit** | **Min** | **Q1** | **Median** | **Q3** | **Max** |
| --- | --- | --- | --- | --- | --- | --- | --- | --- | --- | --- | --- |
| **BCVA** | **Group** | **Eye** | 1.13 | 0.23 | 1.03 | 1.22 | 0.50 | 1.00 | 1.13 | 1.25 | 1.60 |
|  | **Healthy** | **OD** |  |  |  |  |  |  |  |  |  |
|  |  | **OS** | 1.17 | 0.21 | 1.08 | 1.25 | 0.63 | 1.00 | 1.25 | 1.25 | 1.60 |
|  | **MPS** | **OD** | 0.80 | 0.35 | 0.65 | 0.96 | 0.10 | 0.63 | 0.85 | 1.00 | 1.60 |
|  |  | **OS** | 0.80 | 0.35 | 0.65 | 0.95 | 0.20 | 0.50 | 0.80 | 1.00 | 1.60 |
|  | **Fabry** | **OD** | 1.02 | 0.25 | 0.91 | 1.13 | 0.50 | 1.00 | 1.00 | 1.25 | 1.60 |
|  |  | **OS** | 1.06 | 0.21 | 0.96 | 1.15 | 0.63 | 1.00 | 1.00 | 1.25 | 1.60 |
| **SEQ** | **Group** | **Eye** | -0.57 | 1.57 | -1.28 | 0.14 | -5.00 | -1.00 | 0.00 | 1.00 | 1.00 |
|  | **Healthy** | **OD** |  |  |  |  |  |  |  |  |  |
|  |  | **OS** | -0.59 | 1.68 | -1.34 | 0.15 | -5.00 | -2.00 | -0.50 | 1.00 | 2.00 |
|  | **MPS** | **OD** | 3.78 | 3.66 | 1.96 | 5.60 | -2.00 | 1.00 | 3.50 | 5.00 | 13.00 |
|  |  | **OS** | 3.95 | 3.46 | 2.28 | 5.61 | -2.00 | 2.00 | 4.00 | 5.00 | 13.00 |
|  | **Fabry** | **OD** | -0.47 | 3.64 | -2.48 | 1.55 | -12.0 | -1.00 | 0.00 | 2.00 | 3.00 |
|  |  | **OS** | -0.93 | 3.28 | -2.75 | 0.88 | -11.0 | -2.00 | -1.00 | 1.00 | 3.00 |

Table S1: Distribution of BCVA and SEQ by Eye

|  | | | **Mean** | **Std** | **95% CI, lower limit** | **95% CI, upper limit** | **Min** | **Q1** | **Median** | **Q3** | **Max** |
| --- | --- | --- | --- | --- | --- | --- | --- | --- | --- | --- | --- |
| **GAT** | **Group** | **Eye** | 14.06 | 2.96 | 12.81 | 15.31 | 8.50 | 12.25 | 14.00 | 15.75 | 21.00 |
|  | **Healthy** | **OD** |  |  |  |  |  |  |  |  |  |
|  |  | **OS** | 13.39 | 3.38 | 11.99 | 14.78 | 8.50 | 11.00 | 12.00 | 15.50 | 22.00 |
|  | **MPS** | **OD** | 15.34 | 4.63 | 13.29 | 17.39 | 9.50 | 12.30 | 14.50 | 16.00 | 28.70 |
|  |  | **OS** | 15.09 | 5.76 | 12.54 | 17.65 | 8.50 | 12.50 | 14.25 | 16.30 | 38.00 |
|  | **Fabry** | **OD** | 13.18 | 3.35 | 11.66 | 14.71 | 8.50 | 11.30 | 12.00 | 15.00 | 21.00 |
|  |  | **OS** | 13.02 | 3.25 | 11.62 | 14.43 | 6.70 | 11.00 | 13.00 | 15.00 | 19.50 |
| **IOPcc** | **Group** | **Eye** | 14.05 | 3.23 | 12.68 | 15.41 | 8.70 | 11.45 | 13.85 | 15.60 | 22.10 |
|  | **Healthy** | **OD** |  |  |  |  |  |  |  |  |  |
|  |  | **OS** | 15.08 | 3.40 | 13.68 | 16.48 | 7.50 | 13.20 | 15.50 | 17.70 | 21.00 |
|  | **MPS** | **OD** | 15.99 | 9.63 | 11.82 | 20.15 | 6.60 | 11.80 | 14.30 | 16.10 | 55.70 |
|  |  | **OS** | 14.48 | 7.60 | 11.19 | 17.77 | 4.10 | 10.60 | 13.20 | 15.80 | 44.00 |
|  | **Fabry** | **OD** | 15.26 | 5.41 | 12.80 | 17.72 | 8.40 | 12.20 | 14.80 | 17.40 | 32.20 |
|  |  | **OS** | 14.61 | 4.12 | 12.83 | 16.39 | 8.40 | 11.80 | 14.00 | 18.00 | 23.70 |
| **IOPg** | **Group** | **Eye** | 14.57 | 3.48 | 13.10 | 16.04 | 9.00 | 12.40 | 14.35 | 16.00 | 22.50 |
|  | **Healthy** | **OD** |  |  |  |  |  |  |  |  |  |
|  |  | **OS** | 15.83 | 3.92 | 14.21 | 17.45 | 7.90 | 13.50 | 15.10 | 18.40 | 25.70 |
|  | **MPS** | **OD** | 19.03 | 10.86 | 14.34 | 23.73 | 9.90 | 12.00 | 15.80 | 23.50 | 62.30 |
|  |  | **OS** | 18.54 | 8.61 | 14.81 | 22.26 | 8.40 | 14.80 | 16.80 | 19.70 | 52.10 |
|  | **Fabry** | **OD** | 16.51 | 4.43 | 14.49 | 18.53 | 9.30 | 13.30 | 15.60 | 18.90 | 26.70 |
|  |  | **OS** | 15.57 | 4.70 | 13.54 | 17.60 | 8.40 | 12.40 | 14.70 | 18.30 | 26.60 |
| **IOP, palpatory** | **Group** | **Eye** | 13.75 | 1.83 | 12.22 | 15.28 | 12.00 | 12.00 | 13.50 | 15.00 | 17.00 |
|  | **Healthy** | **OD** |  |  |  |  |  |  |  |  |  |
|  |  | **OS** | 14.00 | 1.85 | 12.45 | 15.55 | 12.00 | 12.00 | 14.50 | 15.00 | 17.00 |
|  | **MPS** | **OD** | 16.94 | 2.82 | 15.44 | 18.44 | 12.00 | 15.00 | 16.50 | 20.00 | 20.00 |
|  |  | **OS** | 17.53 | 4.89 | 15.02 | 20.04 | 12.00 | 15.00 | 15.00 | 20.00 | 33.00 |
|  | **Fabry** | **OD** | 14.82 | 3.25 | 12.63 | 17.00 | 8.00 | 13.00 | 15.00 | 17.00 | 21.00 |
|  |  | **OS** | 13.70 | 2.79 | 11.70 | 15.70 | 8.00 | 12.00 | 15.00 | 15.00 | 17.00 |
| **CCT** | **Group** | **Eye** | 541.1 | 27.07 | 529.7 | 552.6 | 503.5 | 521.6 | 531.2 | 569.8 | 589.5 |
|  | **Healthy** | **OD** |  |  |  |  |  |  |  |  |  |
|  |  | **OS** | 540.5 | 27.68 | 529.1 | 552.0 | 494.0 | 526.0 | 542.5 | 555.0 | 590.0 |
|  | **MPS** | **OD** | 557.4 | 98.78 | 514.7 | 600.1 | 409.5 | 507.5 | 543.3 | 599.5 | 840.0 |
|  |  | **OS** | 544.8 | 84.58 | 507.3 | 582.3 | 397.5 | 475.3 | 540.9 | 576.0 | 762.0 |
|  | **Fabry** | **OD** | 564.1 | 31.50 | 549.8 | 578.4 | 503.0 | 549.0 | 563.7 | 588.5 | 615.5 |
|  |  | **OS** | 559.2 | 32.05 | 545.3 | 573.0 | 498.0 | 538.7 | 566.3 | 582.0 | 618.5 |
| **CH** | **Group** | **Eye** | 11.47 | 1.64 | 10.78 | 12.16 | 9.30 | 10.30 | 11.00 | 12.80 | 14.80 |
|  | **Healthy** | **OD** |  |  |  |  |  |  |  |  |  |
|  |  | **OS** | 11.57 | 2.43 | 10.57 | 12.58 | 9.00 | 9.80 | 10.90 | 12.80 | 19.00 |
|  | **MPS** | **OD** | 13.21 | 3.38 | 11.75 | 14.68 | 8.50 | 10.70 | 12.20 | 16.20 | 18.90 |
|  |  | **OS** | 14.22 | 3.98 | 12.50 | 15.94 | 8.90 | 11.10 | 13.70 | 17.10 | 22.30 |
|  | **Fabry** | **OD** | 12.27 | 1.61 | 11.54 | 13.00 | 9.30 | 11.50 | 12.00 | 13.70 | 14.90 |
|  |  | **OS** | 11.73 | 1.55 | 11.06 | 12.40 | 9.10 | 10.80 | 11.70 | 13.00 | 14.80 |
| **CRF** | **Group** | **Eye** | 11.05 | 1.87 | 10.26 | 11.84 | 7.50 | 9.90 | 11.00 | 11.95 | 15.80 |
|  | **Healthy** | **OD** |  |  |  |  |  |  |  |  |  |
|  |  | **OS** | 11.53 | 2.73 | 10.41 | 12.66 | 7.60 | 9.30 | 11.40 | 12.80 | 20.80 |
|  | **MPS** | **OD** | 13.87 | 4.57 | 11.89 | 15.84 | 7.80 | 10.20 | 12.00 | 17.60 | 24.80 |
|  |  | **OS** | 14.57 | 4.73 | 12.53 | 16.61 | 7.00 | 11.00 | 13.70 | 17.60 | 24.20 |
|  | **Fabry** | **OD** | 12.30 | 1.89 | 11.44 | 13.17 | 9.10 | 11.10 | 12.00 | 13.60 | 15.90 |
|  |  | **OS** | 11.90 | 2.81 | 10.69 | 13.12 | 7.50 | 9.90 | 11.60 | 13.60 | 20.70 |
| **Corneal density** | **Group** | **Eye** | 24.93 | 5.95 | 22.42 | 27.45 | 17.10 | 20.75 | 23.50 | 27.60 | 36.80 |
|  | **Healthy** | **OD** |  |  |  |  |  |  |  |  |  |
|  |  | **OS** | 24.49 | 6.64 | 21.75 | 27.23 | 13.40 | 21.40 | 23.20 | 25.80 | 38.00 |
|  | **MPS** | **OD** | 57.90 | 33.68 | 43.33 | 72.46 | 21.00 | 23.30 | 50.50 | 99.00 | 100.0 |
|  |  | **OS** | 56.47 | 31.35 | 42.57 | 70.37 | 20.30 | 26.30 | 43.90 | 87.80 | 100.0 |
|  | **Fabry** | **OD** | 31.38 | 6.65 | 28.35 | 34.41 | 18.80 | 26.50 | 31.80 | 35.80 | 41.50 |
|  |  | **OS** | 31.20 | 8.81 | 27.39 | 35.01 | 18.50 | 24.30 | 31.30 | 36.10 | 48.80 |

Table S2: Distribution of Goldmann applanation tonometry (GAT), corneal compensated intraocular pressure (IOPcc), Goldmann-correlated intraocular pressure (IOPg), palpatory assessed intraocular pressure, central corneal thickness (CCT), corneal hysteresis (CH), corneal resistance factor (CRF) and corneal density

| **Comparison** | **Group** | **Bias** | **95% CI for bias, lower limit** | **95% CI for bias, upper limit** | **Lower Limit of agreement** | **Upper Limit of agreement** |
| --- | --- | --- | --- | --- | --- | --- |
| IOPcc - GAT | Healthy | 0.84 | -0.88 | 2.56 | -6.19 | 7.88 |
| IOPcc - GAT | MPS | 0.22 | -1.64 | 2.07 | -11.71 | 12.14 |
| IOPcc - GAT | Fabry | 1.82 | 0.80 | 2.84 | -4.73 | 8.37 |
| IOPg - GAT | Healthy | 1.48 | -0.47 | 3.43 | -5.47 | 8.43 |
| IOPg - GAT | MPS | 3.45 | 1.58 | 5.33 | -8.62 | 15.53 |
| IOPg - GAT | Fabry | 2.92 | 2.05 | 3.80 | -2.71 | 8.55 |
| Palp - GAT | Healthy | 0.09 | -1.01 | 1.20 | -3.94 | 4.13 |
| Palp - GAT | MPS | 2.35 | 0.95 | 3.76 | -5.42 | 10.12 |
| Palp - GAT | Fabry | 0.57 | -0.70 | 1.85 | -4.90 | 6.04 |

Table S3: Summary of Bland Altman analyses
